# Complete genome sequence data for *Mycobacterium* sp. Panama NTM2, an isolate recovered from a tuberculosis patient in Panama

**DOI:** 10.1128/mra.00026-26

**Published:** 2026-04-02

**Authors:** Joanna E. Rivas Ramos, Fermín Acosta, Priya Patel, Johanna Elizabeth Ku, Indira J. Quintero, Dilcia Sambrano, Julio Jurado, Lizbeth Garibaldi, Mariela Delgado, Odemaris Luque, Jacobus H. de Waard, Luis C. Mejía, Paul R. Johnston, Amador Goodridge

**Affiliations:** 1 Facultad de Medicina and Facultad de Ingeniería, Universidad Latina de Panamá 125801, Panamá, Panamá; 2 Facultad de Medicina, Universidad Interamericana de Panamá 471883, Panamá, Panamá; 3 Instituto de Investigaciones Científicas y Servicios de Alta Tecnología de Panamá 380695, City of Knowledge, Panamá; 4 Sistema Nacional de Investigación, SNI-AIP, Panamá, Panamá; 5 Caja de Seguro Social 217914 , Colón, Panamá; 6 Programa de Control de Tuberculosis, Ministerio de Salud, Colón, Panamá; 7 Instituto de Biomedicina, Universidad Central de Venezuela, Caracas, Venezuela; 8 Escuela de Medicina, Universidad Espíritu Santo, Samborondón, Ecuador; 9 School of Medicine, University of St Andrews 7486, St Andrews, Scotland, United Kingdom; University of Pittsburgh School of Medicine, Pittsburgh, Pennsylvania, USA

**Keywords:** *Mycobacterium tuberculosis*, tuberculosis, bioinformatics, DNA sequencing, nontuberculous mycobacteria

## Abstract

We report the complete genome sequence of *Mycobacterium* sp. strain Panama NTM2, isolated from a sputum sample of a patient with pulmonary tuberculosis in Panama. The 5,922,402-bp genome (68% GC) was assembled into five contigs containing 5,703 protein-coding genes. FastANI identified *Mycobacterium kyogaense* NCTC 11659 as the closest relative (88.13% ANI), suggesting Panama NTM2 represents a novel species within the genus.

## ANNOUNCEMENT

Non-tuberculous mycobacteria (NTM) are a diverse group of Gram-positive, rod-shaped, acid-fast bacilli that are closely related to important obligate human pathogens, such as *Mycobacterium tuberculosis* (1). NTM are usually non-pathogenic; however, they can cause opportunistic infections in an immunocompromised population (2, 3). In this study, we report the complete genome of *Mycobacterium* sp. Panama NTM2, which provides a basis for future functional and comparative genome analysis.

Panama NTM2 was isolated from sputum in Colon, Panama (protocol CBI-USantander-M-024-2023), using Standard-Practical Guidance for Clinical Microbiology methods (4). Briefly, the sputum was incubated in BD BACTEC Mycobacteria Growth Indicator Tube 960 (MGIT) system (37°C, 6 days); acid-fastness was confirmed via Ziehl-Neelsen stain. Pure colonies were recovered on Lowenstein-Jensen culture medium tube and incubated at 37°C for 13 days.

Total genomic DNA was extracted using the ZymoBIOMICS 96 MagBead DNA Kit (Zymo Research, USA) with the addition of an RNase treatment and the resulting DNA eluted in 10 mM Tris, pH 8.5. Long-read libraries (ONT v14 chemistry, SQK-LSK114) were prepared via sequence-independent tagmentation to maintain integrity. Sequencing was performed on an ONT PromethION platform (R10.4.1 flow cells) with Dorado v1.3.0 (5) basecalling algorithm (SUP model) yielding 256,336 raw reads (N50: 1,888 bp; 66× coverage). Short-read libraries (Illumina DNA Prep kit) sequenced on an Illumina NextSeq 2000 platform (2 × 150 bp), yielding 39,097,452 high-quality paired-end reads provided approximately 991× coverage.

ONT read quality was evaluated using raw coverage, read length distributions (N50 and NG50), and mean Phred quality scores (6), with the bottom 5% of lowest-quality reads removed via Filtlong v0.2.1 (7). Illumina reads were processed using fastp v1.0.1 (8) to enforce a minimum quality threshold of Q15 and a length of at least 50 bp while also performing adapter and poly-G trimming.

*De novo* assembly was conducted using a custom pipeline centered on Autocycler v0.5.2 (9), with Flye v2.9.6+ (10) and Hifiasm v0.25.0-r726 (11). For circular genomes, overlaps were trimmed, and the chromosome was rotated to the *dnaA* gene to ensure standard orientation. Plasmids were annotated using Plassembler v1.8.1 (12). Polishing was performed with Polypolish v0.6.0 (13).

Completeness and contamination were assessed using CheckM v1.2.2 (14) and BUSCO v6.0.0 (15) with the mycobacteriales_odb10 lineage, yielding a genome completeness score of 99.1%. The assembly was annotated using the NCBI Prokaryotic Genome Annotation Pipeline (PGAP) v2025-05-06.build7983 (16, 17). Genus-level identity was confirmed using Sourmash (v4.9.4) (18) by comparing the assembly against the GTDB rs226 database. To further resolve species-level classification, whole-genome average nucleotide identity (ANI) was calculated using FastANI (v1.34) (19).

The complete genome of *Mycobacterium* sp. Panama NTM2 comprises five contigs and a genome of 5,922,402 bp long, with a GC% content of 68%. Genome annotation identified 5,703 protein-coding genes, alongside 6 rRNAs, 49 tRNAs, and 3 ncRNAs. The closest relative was *Mycobacterium kyogaense* NCTC 11659 (GCF_003254575.1), with an ANI of 88.13%. Details of the genomic features of Panama NTM2 are presented in Table 1.

**TABLE 1:** Overview of complete genome sequence information of *Mycobacterium* sp. Panama NTM2

Feature	Results of strain
Location	Province of Colon, Republic of Panama
Year	2023
Type	Sputum sample from tuberculosis patient
Length	5,922,402 bp
Number of contigs	5 (1 chromosome + 4 plasmids)
Long-read coverage (ONT)	66.0×
Short-read coverage (Illumina)	991×
GC%	64.3
N50	5,529,317 bp
N90	5,529,317 bp
Coding sequences	5,704
tRNAs	49
ncRNAs	3
rRNAs	6
Completeness %	99.1
Contamination %	0.6

## Data Availability

The complete genome sequence and associated data for *Mycobacterium* sp. Panama NTM2 have been deposited in NCBI under BioProject accession number PRJNA1394949, and the accession for the assembly is JBTHXC000000000. The BioSample accession number is SAMN54340880. The raw Oxford Nanopore read data can be found under the run accession SRR36630062, and the Illumina read data can be found under run accession SRR36630061.

## AFTER EPUB

*[This article was published on 2 April 2026 with errors in the funding. The funding was corrected in the current version, posted on 13 April 2026.]*
